# *Bacillus subtilis-* and *Pseudomonas fluorescens-*Mediated Systemic Resistance in Tomato Against *Sclerotium rolfsii* and Study of Physio-Chemical Alterations

**DOI:** 10.3389/ffunb.2022.851002

**Published:** 2022-04-01

**Authors:** Vaishali Shukla, Sunil Kumar, Yashoda Nandan Tripathi, Ram S. Upadhyay

**Affiliations:** Laboratory of Mycopathology and Microbial Technology, Centre of Advance Study in Botany, Institute of Science, Banaras Hindu University, Varanasi, India

**Keywords:** enzymatic activity, bio-priming, oxidative stress, microbial consortium, *Sclerotium rolfsii*

## Abstract

The present study is a comparative study between Reactive Oxygen Species (ROS) signaling and antioxidative enzymatic signaling and deals with induced systemic resistance (ISR) in enhancing the disease resistance in typical tomato plant (*Solanum lycopersicum* L.) infected by the collar rot fungus, *Sclerotium rolfsii* (Teleomorph: *Athelia rolfsii)* by priming with *Bacillus subtilis, Pseudomonas fluorescens*, and their microbial consortia by a single strain of *Bacillus subtilis*, and *P. fluorescens* as well as by developed microbial consortium with both bacteria. Leaf samples were collected after different durations of pathogen inoculation, i.e., 1, 2, 3, and 4 days, and the systemic level of oxidative stress parameters, such as hydrogen peroxide (H_2_O_2_), photosynthetic apparatus, superoxide radicals, and enzymatic antioxidants, were studied. Plant mortality under various treatments in two different seasons was calculated. The highest H_2_O_2_ was scavenged by the microbial consortium-treated plants (B1P1) and the lowest in pathogen-challenged plants (PC) compared to the untreated control. Cellular damage and reduction in the chlorophyll pigments were the highest at 48 h, and the photosynthetic efficiency (Fv/Fm) was evaluated from 24 to 96 h; the lowest values were observed for pathogen-challenged plants and the highest for B1P1. Enzymatic antioxidants showed the maximum value for B1P1 and the minimum for PC compared to the unchallenged control. Furthermore, an analysis of variance and principal component analysis (PCA) were conducted to examine the effect of the evaluation time (ET) and inoculation conditions (ICs) alone and in combination (ET × IC) on the physiological and biochemical parameters; accordingly, the score and the loading plots were constructed. Tomato root sections inoculated with different treatments were observed through scanning electron microscopy (SEM) to validate the potentiality of primed biocontrol agents in controlling the invasion of the pathogen. Further studies on the potential of this isolate to enhance the plant growth at the field level would strengthen the possibility of using the isolate as an alternative for organic fertilizers and pesticides.

## Introduction

Tomato (*Solanum lycopersicum* L.) is one of the most nutritious and most important crops grown economically g (Abd-El-Kareem et al., 2006). It is considered the best model to study the plant–pathogen interaction. *Sclerotium rolfsii* is a soil-borne polyphagous and a devastating fungus infecting an enormous array of hosts (Sarma et al., 2007), and since tomato is very delicate and vulnerable to *S. rolfsii*, collar rot and eventually wilting occurs on *S. rolfsii* attack. Reactive oxygen species (ROS) and its intermediates (ROIs) are formed in the plant system due to cellular responses; thus, it is considered a possible reason for oxidative stress in the plant systems when they encounter different abiotic and biotic stresses (Thompson, 1996; Ahmad et al., 2010, 2019; Meena et al., 2016; Kohli et al., 2019). The ROS plays an important role in various processes oscillating from plant developmental processes to defense, and it is also helpful for establishing host—pathogen relationships that might be helpful in developing the resistance directly or indirectly against the pathogen in plants (Bandyopadhyay et al., 1999; Mansoor et al., 2022).

Under unfavorable conditions, plants generate a huge number of ROS species involved in the regulation of several processes, including pathogen defense and programmed cell death (PCD) (Huang et al., 2019). However, excessive production of ROS may cause metabolic changes in the host, causing increased damage to proteins, nucleic acids, pigments, and membrane (lipid peroxidation) (Das and Roychoudhury, 2014) with decreased root growth, seed viability, and increased leaf abscission. After a fruitful pathogen invasion and recognition into the host, ROS production is considered the main host cellular reaction that deteriorates the biological molecules. ROS may also play a role in the signaling of pathogenesis-related proteins and in the regulation of genes involved in the accumulation of phenylpropanoid compounds (Punja, 1985; Kiiskinen et al., 2004; Camejo et al., 2016). Therefore, plants regulate the high level of ROS through their antioxidative pool by the release of enzymes, such as ascorbate peroxidase (APX), catalase (CAT), glutathione reductase (GR), superoxide dismutase (SOD), peroxidase (POX), glutathione -S- transferase (GST), and guaiacol peroxidase (GPx), which are ROS scavengers, and several non-enzymatic antioxidants, such as glutathione (GSH), carotenoids, tocopherols, proline, ascorbate, flavonoids, and other phenolics, and thus uphold the homeostatic level of ROS (Ahmad et al., 2019; Kohli et al., 2019; Mansoor et al., 2022). The APX forms a major part of the scavenging pool by catalyzing the reaction of ascorbic acid (AA) with H_2_O_2_, while AA is regenerated through GR (Sewelam et al., 2016). The CAT and APX play an important role in detoxifying ROS and catalyzes the conversion of peroxide molecules into water and oxygen (Das and Roychoudhury, 2014). Oftentimes, these defensive mechanisms are superseded by the overproduction of ROS during a pathogenic attack which causes disease advancement. Thus, to compensate this, seed priming is one such strategy to develop resistance against various stresses, including ROS generation (Singh et al., 2013a; Künstler et al., 2015). Recent studies presented that the induced systemic resistance (ISR) of *Bacillus* sp. is associated with the activation of plant defense mechanisms (Kloepper et al., 2004; Van Loon, 2007; Li et al., 2015). *P. fluorescens* is a well-known plant growth-promoting rhizobacteria (PGPR), inducing the resistance mechanism in the host plant through a range of exogenous molecules. *P. fluorescences* helps in improving the biotic stress by inducing ISR in the host plants (Kumudini et al., 2017). However, studies on the detailed understanding of the oxidative stress alleviation mechanism through *Pseudomonas*-priming are limited.

In the current experiment on *Bacillus subtilis* and *P. fluorescences* biocontrol agents (BCAs) were used alone and with microbial consortium against the invading pathogen to check their potential for plant growth and development as well as disease management (Hammerschmidt et al., 1982; Sharma et al., 1998). The present study, observed the use of rhizosphere microbes (*P. fluorescences* and *B. subtilis*) individually and in combinations to control the collar rotting disease and the dynamics of metabolic changes (physiological and biochemical) in tomato plants against *S. rolfsii* by using the PGPR priming method. *P. fluorescences* and *B. subtilis* bacteria are well-known BCAs that alleviate the biotic stress in the host plants by inducing ISR, and a Principal Component Analysis (PCA) was performed to observe the effect of time, treatments, and both together for the various parameters/variables used.

## Materials and Methods

### Host and Growth Conditions

Susceptible varieties of tomato seeds (Selection −7) were obtained from the Indian Institute of Vegetable Research (IIVR), Varanasi, India. The plants were potted in sterilized soil in a glasshouse with a duration of 13 h light and 11 h dark photoperiod at 27 ± 0.5°C. The seeds were surface sterilized with 1% sodium hypochlorite (NaOCl) for 30 s, followed by ethanol, and finally washed twice with sterilized distilled water (SDW) to remove traces of NaOCl and then grown in plastic pots (15 cm × 10 cm).

### Collection and Culture of Bacterial Isolates

Biocontrol agents used *Bacillus subtilis* BHU M (MF 919590), which was obtained from the culture collection from the laboratory of Mycopathology and Microbial Technology and *P. fluorescences* BHU V (NAIMCC-B-0036) from the National Agriculturally Important Microbial Culture Collection, ICAR-National Bureau of Agriculturally Important Microorganisms, Mau Uttar Pradesh, India (NBAIM). *B. subtilis* BHU M and *P. fluorescences* BHU V were grown on nutrient agar (NA) in an incubator for 24–48 h at 30°C.

### Collection and Culture of Fungal Isolate

*Sclerotium rolfsii* BHU S (NAIMCC-F-01643) was obtained from the NBAIM, Mau, Uttar Pradesh. The sclerotia were surface sterilized using 1% of NaOCl, followed by ethanol and finally rinsed twice with SDW to remove NaOCl traces and placed on potato dextrose agar (PDA) amended with streptomycin for 4–5 days at 28°C in light/dark for 12 h in a light/dark photoperiod.

The pathogen was initially identified using morphological characteristics, such as mycelia structure, sclerotium formation, shape, size, color, and pattern, under a compound microscope at 40 times following standard manuals (Ellis, 1976; Simmons, 2007), and further confirmed by the amplification of the Internal transcribed spacers (ITS) using the universal primers, ITS1 and ITS 4 amplifying the ITS regions and 5.8S genes. The DNA extraction was carried out as per the method of Doyle and Doyle (1987). The lyophilized micellar mats of 0.5 g were ground with a mortar and pestle, using 10 ml of Cetyl trimethyl ammonium bromide (CTAB) extraction buffer, and then incubated at 65°C in a water bath for 30 min. The sample was then mixed with an equal volume of chilled chloroform/isoamyl alcohol and gently mixed, followed by centrifugation at 10,000 rpm for 10 min at a temperature of 4°C. The supernatant thus obtained was mixed with an equal volume of isopropanol and left for 2 h at 4°C. The sample was again centrifuged at 10,000 rpm for 10 min at a temperature of 4°C. The pellet was then rinsed with 70% ethanol and air-dried for 4 h to remove the traces of alcohol. The amplification ITS rDNA reaction was performed in 25 ml reaction mixture containing 2.5 ml of 10 times reaction buffer, 5 ml of each deoxyribonucleotide triphosphate (dNTP), 1.0 ml of each of ITS and 5.8S region-specific universal ITS 4 and ITS 1 primers, 0.3 ml of Taq DNA-polymerase, and 10–100 ng of DNA and 2.5 ml of MgCl_2_. The optimized thermal profile of PCR was initially denaturated at 95°C for 3 min, denaturated at 95°C for 30 s, annealed at 70°C for 30 s and finally extended at 72°C for 1 min with additional 40 cycles. The amplification was confirmed on a 1% agarose gel in 0.5 × Tris-borate-EDTA (TBE) buffer and visualized under a UV-transilluminator.

### Pathogenicity Test

A pathogenicity test was performed following Kosch's postulate. Accordingly, the tomato seeds were sterilized in 1% of NaOCl for 30 s, and then subsequently with 70% of ethanol and finally splashed 2–3 times with sterilized distilled water. The seeds were sown in autoclaved soil and after 20–25 days of sowing, at the 3–4 leaf stage, the plants were inoculated with pure cultured pathogen at the collar region maintaining the physiological temperature and humidity of 27–30°C and 60–70%, respectively. After 48 h, the visible symptoms began to appear near the collar region, and leaf yellowing was also seen. After 96 h, complete wilting and dark brown round sclerotia were observed. The collar region and sclerotia were cut and washed in running water, followed by sterilizing the plant part as described previously. The freshly poured PDA plates were inoculated with collar part and sclerotia and kept at 25–27° C in an incubator. The plates were observed after 2–3 days.

### Inoculum Preparation in Plants, Microbial Association, and the Experimental Design

Inoculum of *B. subtilis* and *P. fluorescences* was primed for all experiments by harvesting the cells from nutrient broth cultures grown at 30 ± 1°C for 48 h followed by centrifugation at 6,000 rpm for 25 min. The inoculum was re-suspended in sterile distilled water, after which the concentration was adjusted using a spectrophotometer to 10^8^ CFU/ml. These suspensions were further used for the seed treatment method (Silva et al., 2003). Selection-7 cultivar of tomato seeds (8 seeds per pot and 5 plants per treatment) were surface sterilized using 1% of NaOCl followed by washing with ethanol and finally twice with SDW to remove traces of NaOCl. The seeds were dried in laminar flow for 4–5 h. The seeds were primed with *Bacillus* and *Pseudomonas*.

**Table:** 

Control	CNT
Pathogen-challenged plants	PC
PC+ *Bacillus subtilis*	B1
PC+ *P. fluorescens*	P1
PC+B1+P1	B1P1

Seeds were treated with BCAs singly inoculated and with a microbial consortium. Dried tomato seeds were put into a different combination of treatments along with 1% of carboxymethylcellulose (CMC). The potting mixture contained sandy soil and vermicompost manure in a 2:1 ratio and was sterilized at 15 psi for 25 min in an autoclave. Plastic pots with a dimension of 15 cm × 10 cm were used and kept in a glasshouse and were monitored regularly. In the following 4 weeks, the plants were treated with *S. rolfsii*, which were mass cultured on bajra (*Pennisetum typhoides*) seeds (bajra seed 150 g, sand 450 g, distilled water 150 ml) for 15 days at 27 ± 2°C (Singh et al., 2013b). All the treatments were performed in triplicates and after 24 h of post-pathogen inoculation, the leaves were harvested at an interval of 24, 48, 72, and 96 h for various assays. The disease incidence was calculated by the number of infected plants/Total number of plants assessed × 100 (Chakraborty et al., 2019).

### Evaluation of Photosynthetic Parameters

#### Photosynthesis Measurements

The chlorophyll fluorometer (PAM, Walz Germany) was used for the measurement of the Fv/Fm ratio which indicates the photosynthesis efficiency. The method of Ramezani et al. (2017) was adopted for the study. Accordingly, the potted plants were dark-adapted for 15 min at room temperature before the measurement. The middle of the leaf lamina from the plant which was dark-adapted for 15 min, was stuck in a leaf-clip diode, and light of an intensity of 3–4 μ mol m^−2^ s^−1^ was allowed to pass to measure the initial fluorescence (F_o_), and for the maximum and final fluorescence (Fm), an exposure of saturating white light of 4,000 μmol m^−2^ s^−1^ was provided. Fv which is the variable fluorescence was calculated by subtracting Fo from Fm (Fv = Fm–Fo) and thus, Fv/Fm was determined.

#### Chlorophyll Content Estimation

A total of 0.1 g leaves from every treatment were crushed in 80% of acetone, followed by centrifuging at 12,000 × g for 10 min at 4°C, and the supernatant was taken for the estimation of chlorophyll a, b, carotenoids, and total chlorophyll at 645, 663, and 470 nm, respectively.

Chlorophyll a and b, and total chlorophyll calculated according to the study by Lichtenthaler (1987) are as follows:


$$\begin{array}{l} \text{Chlorophyll a (mg/g leaf fresh weight)}\\ \;=\left[12.7\left(OD\;663\right)-2.69\left(OD\;645\right)\right]\times V/1,000\;\times \;W\\ \text{Chlorophyll b (mg/g leaf fresh weight)}\\ \;=\left[22.9\left(OD\;645\right)-4.68\left(OD\;663\right)\right]\times V/1,000\;\times \;W\\ \text{Total Chlorophyll (mg/g leaf fresh weight)}\\ \;=\left[20.2\left(OD\;645\right)-8.02\left(OD\;663\right)\right]\times V/1,000\;\times \;W \end{array}$$

Where, OD = Optical Density, V = Volume of sample, and W = Weight of sample.

To envisage the photosynthetic efficiency under various pathogen stresses alone and with biocontrol agents, a radar diagram was made to see various photosynthetic parameters (chlorophyll a, chlorophyll b, total chlorophyll, carotenoid, and Fv/Fm) of two different consecutive seasons (July to Nov and Nov to Feb). Additionally an action spectrum was constructed for various treatments at 48 h.

#### Biochemical and Physiological Profiling

Nodal leaves (3rd to 5th nodes) from every replicate of each treatment, were plucked from the bottom from 24 to 96 h of pathogen inoculation. Leaves were then washed in running water twice with the utmost care, dehydrated with blotting paper, and deep-frozen (−80°C) for further biochemical analysis.

#### Detection of Hydrogen Peroxide Accumulation

The histochemical 3,3′-Diaminobenzidine (DAB) staining method was employed to see the accumulation of H_2_O_2_ as described by Thordal-Christensen et al. (1997) with few modifications. For infected as well as control leaves, after 48 h, pathogen inoculations were taken. Leaves were cut with a sterilized knife 1 cm above the petiole and put down into a beaker containing 1 mg/ml DAB-HCl (pH 5.6). The beaker was kept in a humid chamber for 8 h in dark. The produced H_2_O_2_ reacts with DAB to form a horseradish brown-colored stain over the laminar region of the leaf; it was removed *via* boiling it into a solution of 70% ethanol so as to see the accumulation of H_2_O_2_ in the treated samples as well as the control. Pictures of the leaves were taken with a Nikon DSLR camera D3500 as well as using a light microscope (Zeiss Nikon, USA) for microscopic visualization.

#### Quantification of H_2_O_2_

The quantification of H_2_O_2_ was performed following the method of Patel et al. (2017) with little modification. In total, 0.2 g of leaf from each treatment was taken and crushed in 2.0 ml of 0.1% (w/v) trichloroacetic acid (TCA) in an ice bath. The homogenate was centrifuged at 10,000 × g for 20 min, and 0.5 ml of supernatant was taken for further analysis. To the supernatant, 10 mM of potassium phosphate buffer (pH 7.0) and 1 ml of potassium iodide was added, kept for 5 min, and then spectrophotometrically measured at 390 nm. The amount of H_2_O_2_ formed was measured from the standard curve and expressed as n mol H_2_O_2_ g^−1^ fresh weight (FW).

#### Ascorbic Acid Estimation

Ascorbic acid was determined according to the method of Klein and Perry (1982) with some modifications. Tomato leaves (0.5 g) were extracted with 10 ml of 1% metaphosphoric acid followed by shaking at 500 rpm for 30 min at room temperature and filtered through Whatman No. 4 filter paper. The filtrate was centrifuged at 10,000 rpm for 20 min. The supernatant (1 ml) was mixed with 9 ml of 2,6-dichlorophenolindophenol (DCPIP) and the absorbance of the solution was measured within 30 min at 515 nm against a blank. The AA content was calculated on the basis of the standard curve, which was prepared with a series of the known AA solutions added with 3% (w/v) of metaphosphoric acid. The results were expressed as mg ascorbic acid g^−1^ FW of tomato. The experiment was replicated with three independent assays.

### Determination of ROS Production

#### Detection of ${\text{O}}_{2}^{-}$ Accumulation via Histochemical Staining

Treated and control leaves kept for 48 h were immersed in 50 mM of potassium phosphate buffer (pH 7.8) consisting of 0.1% nitroblue tetrazolium (NBT) and 10 mM of sodium azide (NaN_3_) for 2 h in the dark, and were then immersed in 75% v/v of ethanol to completely remove the chlorophyll. The formation of purple formazan over the laminar and petiolar regions indicated the production of superoxide radicals in the leaves.

#### Superoxide Dismutase (SOD, E.C. 1.15.1.1) Assay

Superoxide dismutase activity was performed following the method of Beauchamp and Fridovich (1971) with few modifications. For this, 0.1 g of leaf was homogenized in 5 ml of ice-cold extracting solution containing 0.1 M of phosphate buffer (pH 7.5) and 0.5 mM of ethylenediamine tetraacetic acid (EDTA), which was then further centrifuged at 13,000 × g at 4°C for 25 min. The 3 ml reaction mixture consisted of 50 mM of phosphate buffer (pH 7.8), 13 mM of methionine, 60 μM of riboflavin, 0.1 mM of EDTA, and 75 μM of NBT, to which 100 μl of enzyme extract was added. The reaction was started by keeping the tubes under fluorescent lamps until the NBT in the mixture was reduced, shown by a changing color. Absorbance of the amount of SOD caused 50% inhibition of NBT, which was spectrophotometrically measured at 560 nm and expressed in terms of U SOD min^−1^ mg^−1^ protein.

#### Phenylalanine Ammonia-Lyase (PAL, E.C.4.1.3.5) Assay

The assessment of PAL was determined according to the protocol of Brueske (1980) with few modifications. About 0.2 g of leaf was crushed in ice-cold 0.1 M of sodium borate buffer (pH 7.0) mixed with 1.4 mM of mercaptoethanol; then subsequently centrifuged at 15,000 × g at 4°C for 20 min. A total of 0.2 ml of enzyme extract was added to a reaction mixture containing borate buffer (pH 8.7) and distilled water. To initiate the reaction, 0.1 mM of *L*-phenylalanine (0.5 ml, pH 8.7) was added to each test tube and then incubated for 30 min at 30°C. About 1 M, 0.5 ml of TCA was used to cease the reaction and absorbance against the blank (sodium borate buffer pH 7.0), was recorded at 290 nm and the quantity of PAL activity was calculated in terms of μ mol TCA min^−1^ mg^−1^ protein.

#### Peroxidase (POX, EC 1.11.1.7) Assay

Peroxidase activity was determined using the method of Gill and Tuteja (2010) with few modifications. A total of 0.2 g of leaf samples from different treatments were homogenized in 2 ml of 0.1 mol l^−1^ phosphate buffer (pH 7.0) in a cold chamber (4°C), centrifuged at 13,000 × g for 30 min at 4°C, and the supernatant was used as an enzyme source. The reaction mixture consisted of 0.05 ml of enzyme extract, 0.5 ml of H_2_O_2_ (1% v/v), and 1.5 ml of pyrogallol (0.05 mol l^−1^). For control, the reaction mixture without the enzyme extract was taken. The change in the absorbance was recorded at 420 nm after 30 s intervals up to 3 min. Enzyme activity was expressed as a change in μ mol min^−1^ mg^−1^ protein.

#### Catalase (CAT, EC 1.11.1.6) Assay

Catalase activity was analyzed using the protocol of Aebi (1984) with few alterations. Leaf samples (0.1 g) were homogenized in a buffer consisting of 50 mM of Tris-HCl (pH 8.0) in a pre-chilled mortar and pestle at 4°C in a cold room chamber. The buffer solution consisted of 0.5 mM of EDTA, 2% w/v of polyvinylpyrrolidone (PVP), and 0.5 % v/v of Triton X 100, which was then centrifuged at 13,000 × g at 4°C for 20 min to use the obtained supernatant for enzymatic assay. To 300 μM of phosphate buffer (pH 7.2) and 100 μM of H_2_O_2_, 1 ml of enzyme extract was added and kept in the dark for 1 min to measure the total oxygen evolved from the dissociation of H_2_O_2_, and measured in n mol min^−1^ mg^−1^ protein.

#### Ascorbate Peroxidase (APx, EC 1.1.11.1) Assay

The method of Mandal et al. (2008) was followed for the estimation of APX with few modifications. The reaction mixture consisted of 0.2 ml of enzyme extract, 0.25 mM of AA, 25 mM of phosphate buffer (pH 7.0), 0.1 mM of EDTA and 1.0 mM of H_2_O_2_. The APX activity was spectrophotometrically measured at 290 nm by recording the decrease in absorbance after the addition of enzyme extract for 2 min. The activity of APX was presented as nmol ascorbate oxidized min^−1^ mg^−1^ protein.

#### Glutathione Reductase (GR, EC 1.11.1.6) Assay

A total of 0.1 g of the leaf sample was crushed in a pre-chilled mortar pestle in 5 ml of 50 mM Tris-HCl buffer (pH 7.6). The homogenate was centrifuged at 13,000 × g at 4°C for 35 min and the obtained supernatant was taken for further analysis. The supernatant was added to the reaction mixture comprising of 50 mM of Tris-HCl buffer (pH 7.6), 10 ml of NADPH (0.15 mM), 100 μl of oxidized GSH (1 mM GSSG), 3 mM of MgCl_2_, and 0.3 ml of enzymatic extract, and all the experiments were performed in a 4°C cold chamber following the method of Anderson (1996) with few modifications. The GR activity was measured spectrophotometrically at 340 nm by observing the decrease in the absorbance of NADPH, and the enzyme activity was expressed as μ mol min^−1^ mg^−1^ protein NADPH oxidized. Protein estimation was done using the method of Bradford (1976), and standard curves were prepared by bovine serum albumin (BSA; sigma) reagent.

#### Root Colonization Efficiency of the Strains Against *S. rolfsii* by SEM Analysis

Twenty-five-day-old tomato plants untreated (control), positive control (PC), and bacterial strain-inoculated plants (B1, P 1, B1P1) were treated with pathogen and after 48 h, they were gradually lifted from pots and the soil particles surrounding the roots were removed and cut into 2–3 mm of thick fragments. The root samples were washed with water for removing the soil particles and fixations were carried out by using 2.5% glutaraldehyde in phosphate buffer at 4°C for 15 min. After fixation, the samples were osmicated and dehydrated through an ascending series of graded ethanol (30, 50, 70, 80, 90, and 95% for 15 min and 100%, two times for 15 min for each sample) at 4°C. Then the sample was proceeded to final steps by critical point drying, and sputter coated with Au-Pd. The root colonization status by bacterial strains was investigated with the help of an SEM (FEI. Quanta 250).

### Statistical Analyses

Experiments performed were completely randomized and done in replicates. All the data are represented as mean ± standard error of the mean for autonomous experiments of various treatments. A one-way ANOVA was performed using Tukey's B test (*p* ≤ 0.05) and a *post-hoc* test was also done to see significant differences among the different groups through SPSS (IBM SPSS Statistics version 16) software. A PCA was performed using Minitab software so as to determine the relationship between various parameters and variables used in the experimentation.

## Results

**Figure 1:**
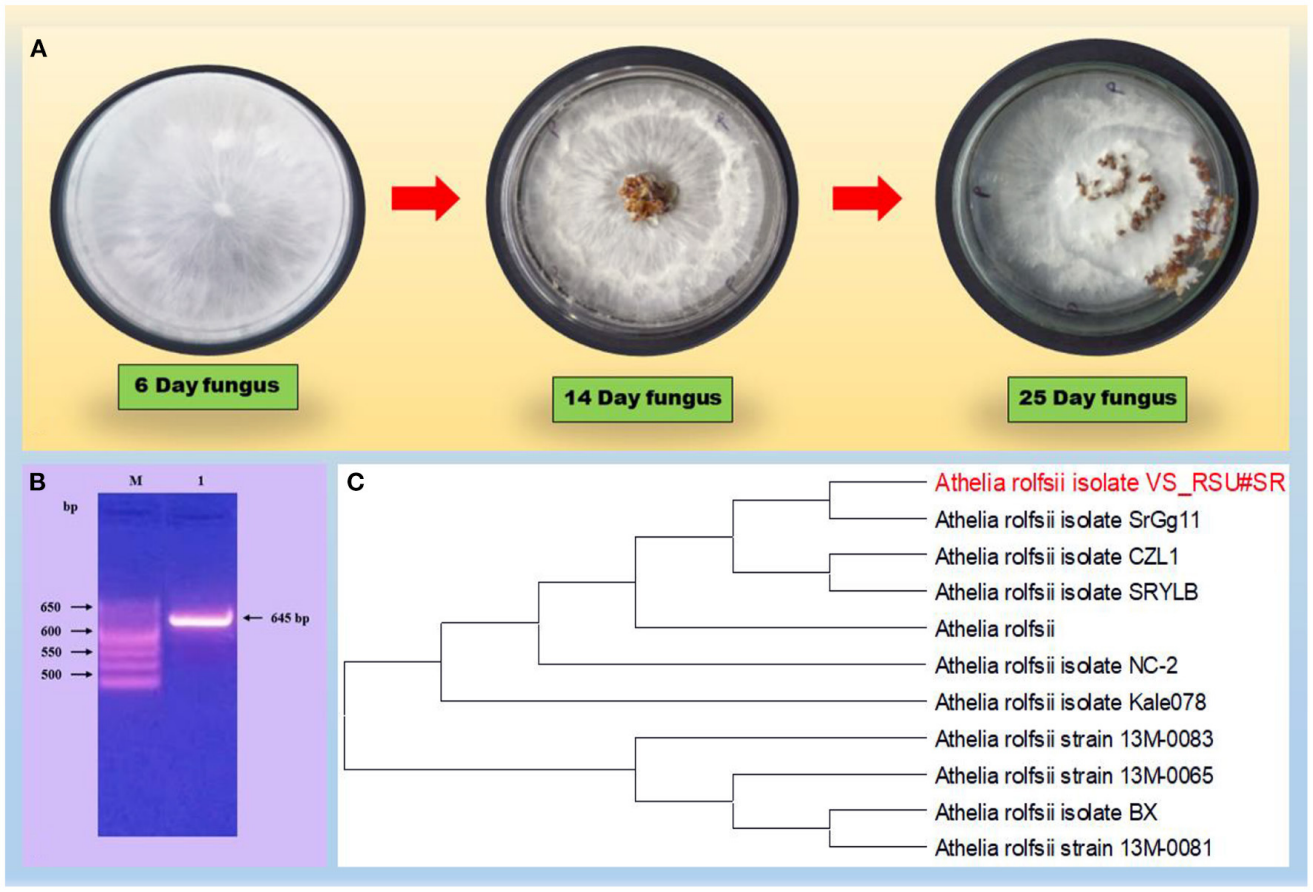
**(A)** Describes the morphology of the fungus on PDA plate. **(B)** Gel electrophoresis of the PCR product with primers, ITS1/ITS4 of DNA from the fun-gal isolate. Lane M, molecular weight markers (1 kb ladder); lanes 1, Athelia rolfsii (645 bp); **(C)** Phylogenetic tree on a fungal strain, the Athelia rolfsii isolate, based on the 18S rDNA gene sequence. A boot strap consensus tree was drawn by multiple sequence alignment with a method using neighbor-joining software, MEGA X.

Based on the preliminary microscopy examination, *S. rolfsii* was identified on seeing the morphology of the fungus. The formation of small, round, and dark brown sclerotia on the PDA plate was further confirmed on the basis of universal primers and the 5.8S gene having some regions specific for the pathogen. The amplicon size was found at 645 bp, which is a characteristic of *A. rolfsii* (Figure 1). ITS sequencing was made using the universal primers, ITS1 (forward) and ITS4 (reverse) and the r DNA sequences thus obtained were analyzed using NCBI-BLAST. The BLAST results confirmed the species as *A. rolfsii*. The sequence was submitted to a gene bank and the accession number MW835230 was assigned for the identified isolate.

### Pathogenicity Test

**Figure 2:**
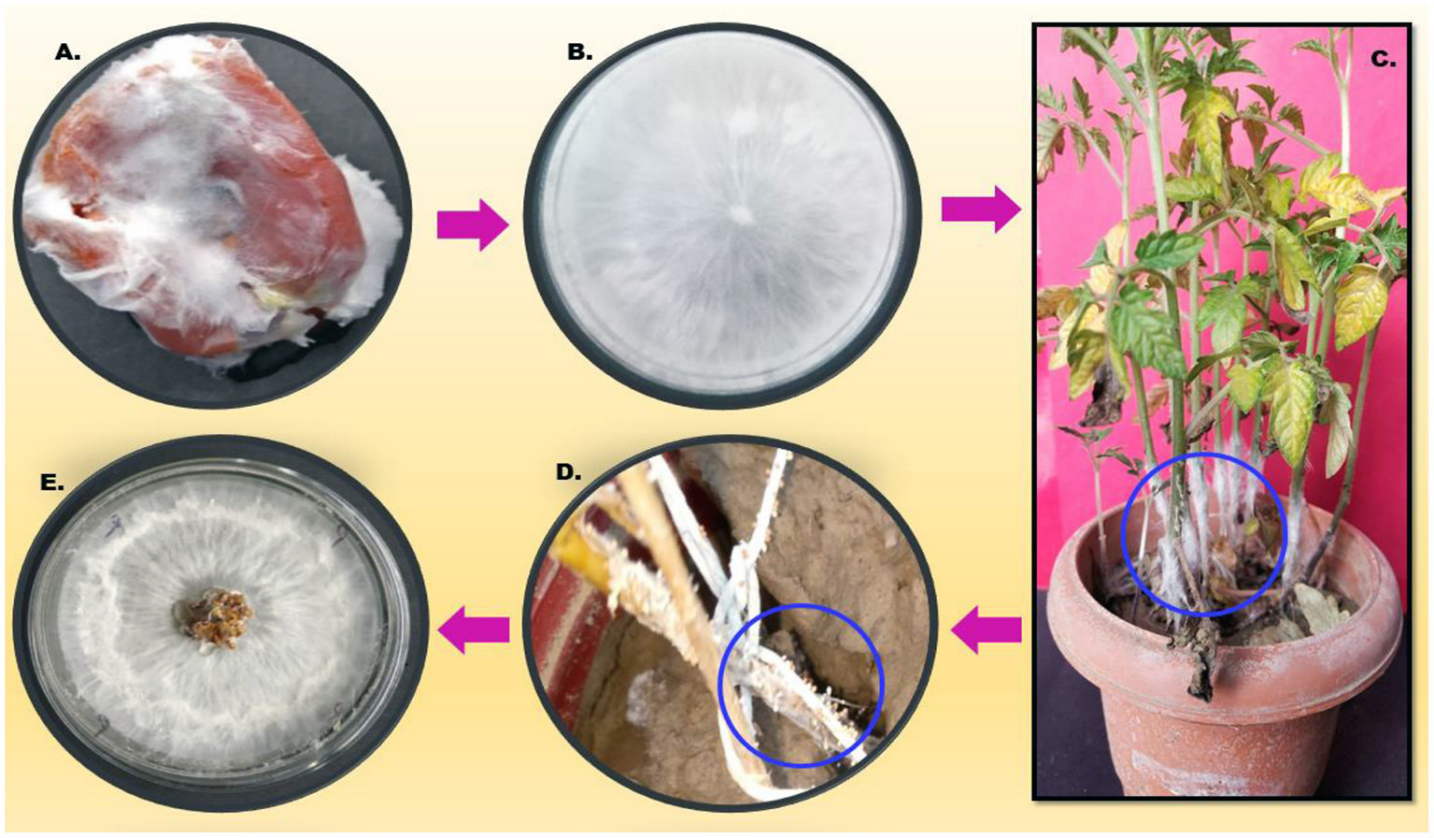
Describes the pathogenicity cycle. **(A)** Infected tomato; **(B)** Inoculation on the PDA plate; **(C)** pathogen inoculation on the host; **(D)** sclerotia formation on the stem of the infected host; and **(E)**. reinoculation on the fresh PDA plate.

After 24 h of post-pathogen inoculation, visible disease symptoms were observed. After 48 h, rotting was observed near the collar region and leaf yellowing was seen, and after 96 h, full wilting of the plant was observed. The infected part was reinoculated on a PDA plate and observed morphologically under a microscope, which resulted in a similar type of morphology (Figure 2).

### Plant Microbial Association and Disease Incidence

**Figure 3:**
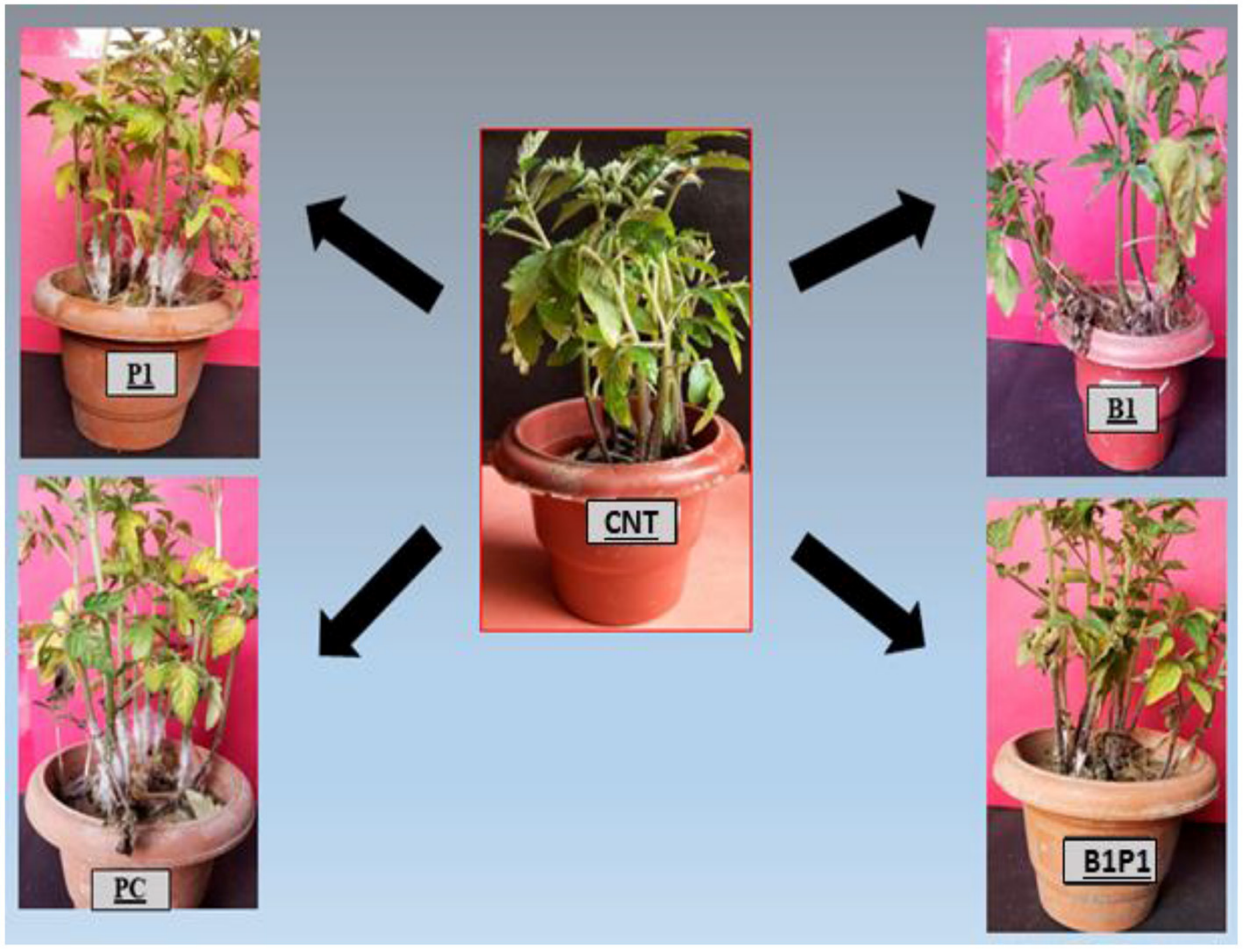
Represents the effect of different BCAs against the virulent phytopathogenic fungus, *S. rolfsii* on 25-day old tomato plants that were planted under a glasshouse temperature and humidity condition. The results showed that BCAs, when used in consortia (B1P1), showed the lowest plant mortality, i.e., 15%, followed by singly inoculated and the highest plant mortality was observed in the positive control (PC), i.e., 84%.

The plants were screened for the disease occurrence after 24 h post-pathogen inoculation and after 72 h, maximum mortality was recorded, and the mortality percentage was calculated (Figure 3). The highest mortality was recorded in pathogen-challenged (PC) plants and the lowest mortality was recorded in the triple microbial consortium-treated plants (B1P1).

### Evaluation of Photosynthetic Apparatus

#### Photosynthesis Measurements

**Figure 4:**
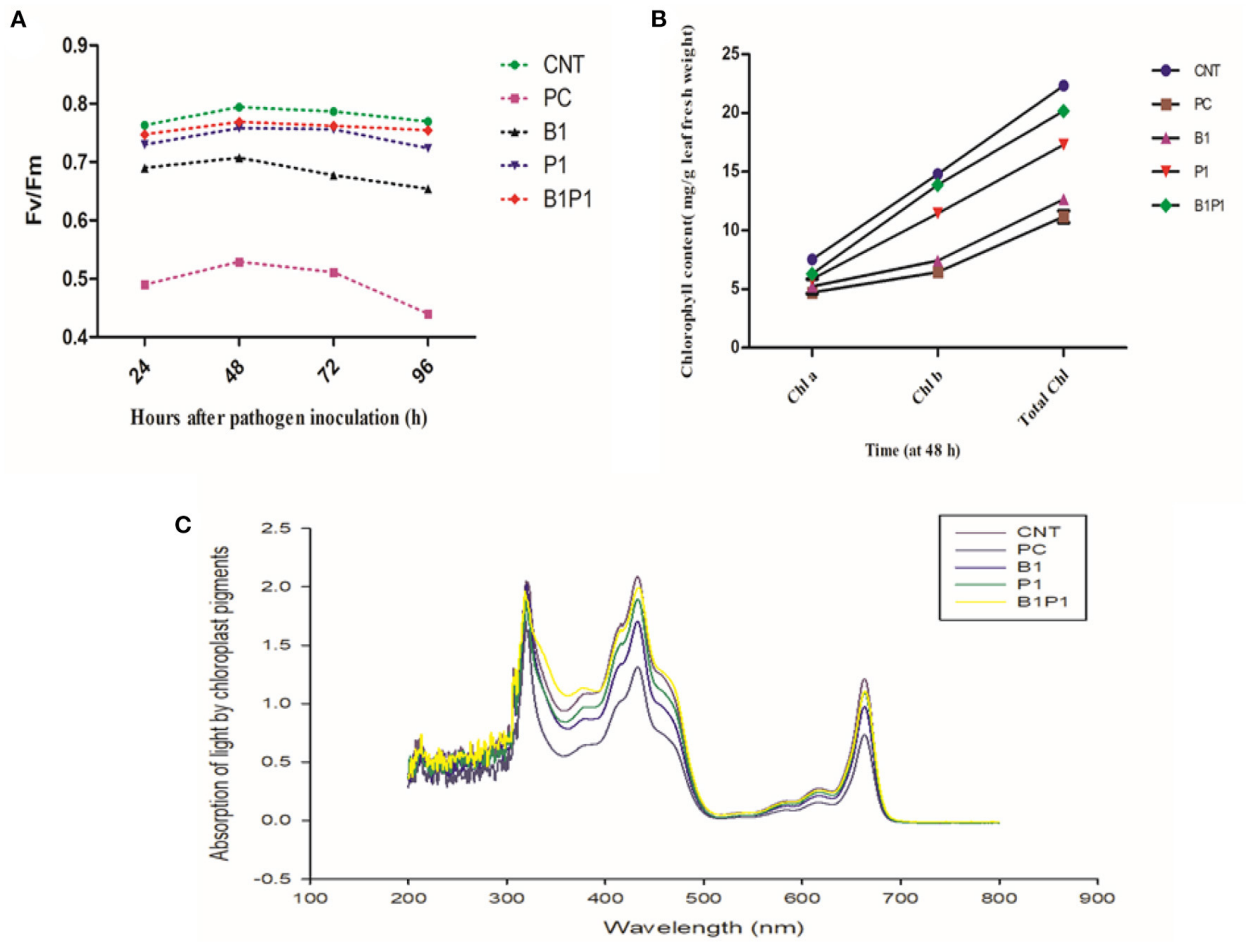
Impact of different treatments on **(A)**. Photosynthetic efficiency (Fv/Fm); **(B)** Chlorophyll content (Chl a, Chl b, Total Chl) on tomato leaves at 48 h of pathogen inoculation; **(C)** Absorption spectrum against wavelength (nm) at 48 h. Data shown as a mean ± SE (*n* = 3). Tukey's B *post-hoc* test at 5% confidence level (*p* < 0.05) was used to see the significance of data.

Chlorophyll fluorescence quenching parameter, Fv/Fm was studied against various treatments for different hours of pathogen inoculation i.e., 24, 48, 72, and 96 h, and the maximum efficiency was observed at 48 h for different treatments. The highest Fv/Fm values were found for B1P1 followed by P1 and the lowest were found for PC against the control (Figure 4A).

#### Chlorophyll Content Estimation

The effect on chlorophyll parameters was observed for 48 h for different treatments. The chlorophyll content was highest in B1P1, followed by single, and the lowest in pathogen-challenged (PC) plants compared to the control (Figure 4B). Following the formulae described by Lichtenthaler (1987), for chlorophyll a, the lowest reduction was found in B1P1, of 16.33%, followed by P1, of 18.69%, and the highest was found in PC, of 37.56%, compared to the untreated control. On examining the chlorophyll b content, the trend was found to be similar, i.e., the lowest reduction in B1P1, of 6.22% followed by P1, of 15.48%, and the highest in PC, of 56.38%. In total chlorophyll, B1P1 was found to have the lowest reduction, of 9.62%, followed by P1, of 16.61%, and the highest reduction in PC, of 50%.

**Figure 5:**
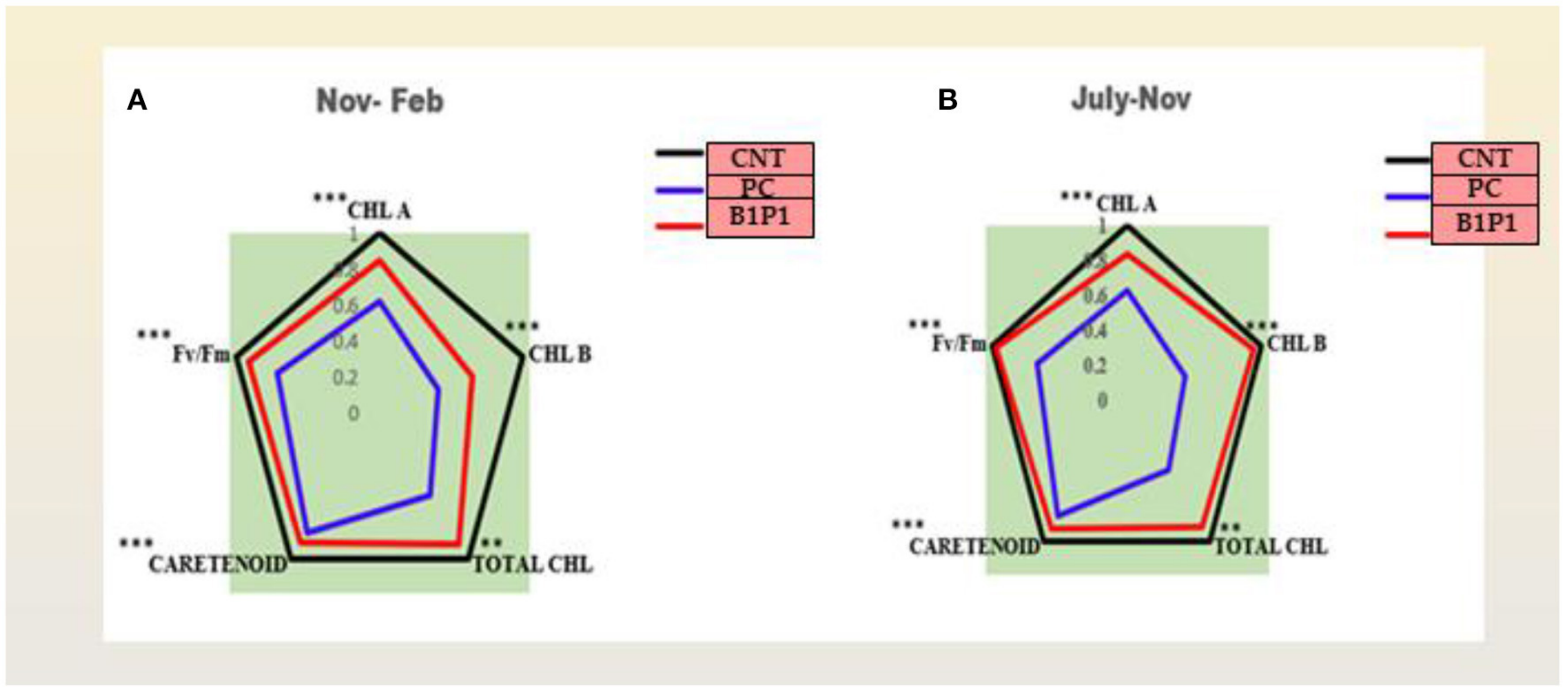
**(A,B)** Effect of different treatments on chlorophyll a, chlorophyll b, total chlorophyll, carotenoids, and Fv/Fm in two different consecutive seasons. Radar charts depict changes in the relative content of the pigments in three treatments in two seasons. The relative content was evaluated as a ratio of each content of pigment to its control. Values are calculated as a mean of *n* = 3. Asterisks represent the significance (***significant at *p* < 0.01, **significant at *p* < 0.001) using a one-way ANOVA.

The action spectrum explained the peaks of chlorophyll a and b, which showed the highest value in the control followed by B1P1, P1, and PC (Figure 4C). A radar chart was constructed for better visualization of distinct photosynthetic pigments (Chl a, Chl b, Total chl, and carotenoids) and photosynthetic efficiency (Fv/Fm); moreover, the relative content was measured as a ratio of each pigment in plants treated with the pathogen effect alone (PC) and in the microbial consortium-treated plants with biocontrol agents (B1P1) to its control (Figure 5) for two different seasons. Comparing both the seasons, November to February showed a more positive response to various treatments than July to November. The size of the pentagons, i.e., the area covered, decreased more in PC plants than in the microbial consortium-treated plants compared to the control in both the seasons.

### Biochemical and Physiological Profiling

#### Detection of Hydrogen Peroxide Accumulation

**Figure 6:**
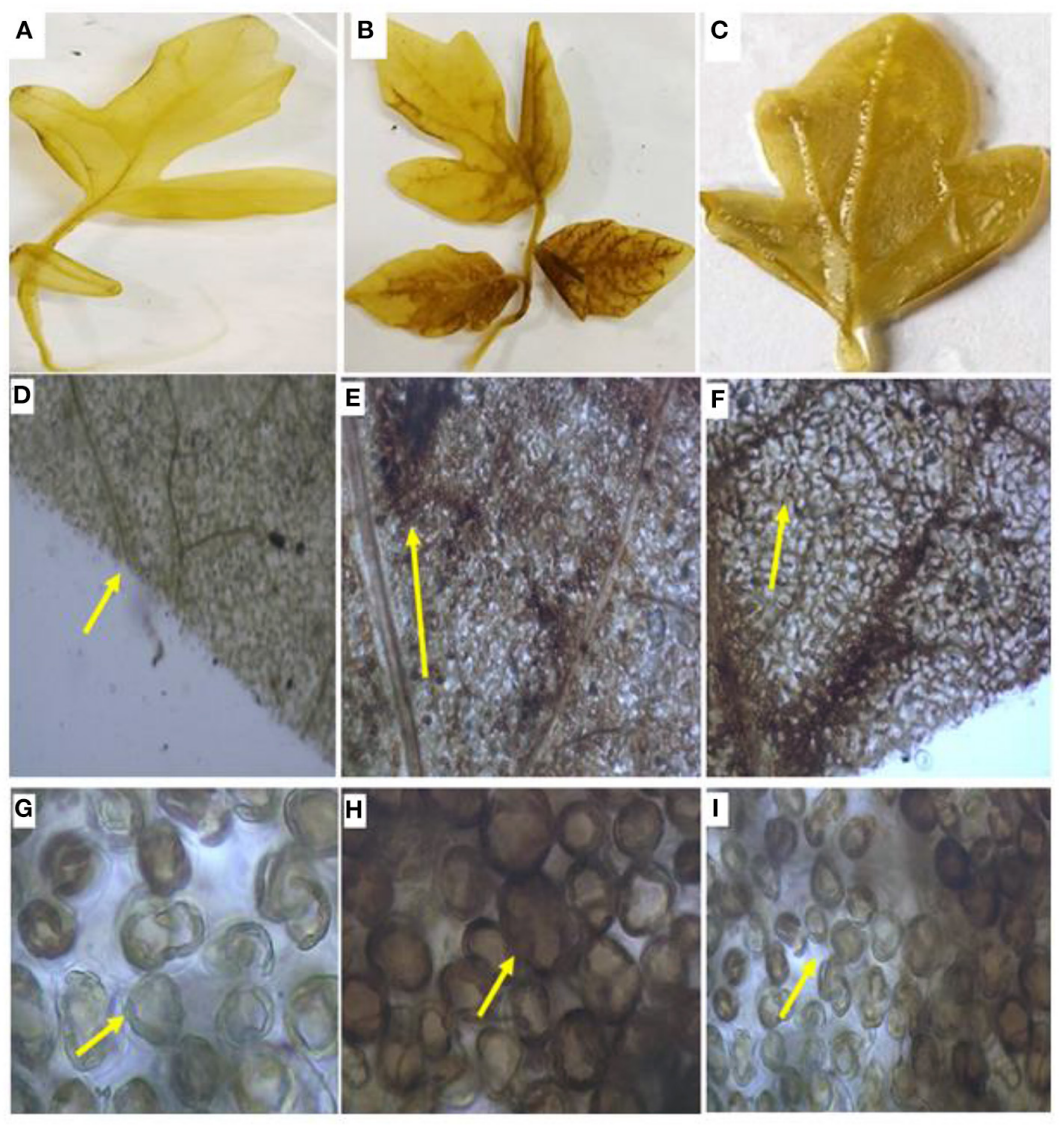
The H_2_O_2_ content in the tomato leaves. **(A)** Control leaf, **(B)** PC leaf**; (C)** B1P1 leaf; **(D)** Control leaf under 10 times magnification, **(E)** PC leaf under 10 times magnification; **(F)** B1P1 leaf under 10 times magnification; **(G)** Control leaf under 40 times magnification, **(H)** PC leaf under 40 times magnification, **(I)** B1P1 leaf under 40 times magnification (yellow arrows indicate deposition of peroxide radicals in the leaf and around cells).

Hydrogen peroxide (H_2_O_2_) was qualitatively visualized with DAB staining, which is a benzene derivative, after 48 h. Upon pathogen treatment, the development of brown precipitate of horse reddish peroxide (HRP) indicates oxidation of H_2_O_2_ in the treated area. The highest oxidation occurred in the pathogen-challenged (PC) plants and the lowest in B1P1 compared to the control in the laminar zone. The microscopic visualization was also examined, and the images were obtained at 10 times and 40 times, respectively (Figure 6). Histochemical staining using DAB showed the highest accumulation of H_2_O_2_ in petiolar region, and also in the interveinal regions after 48 h in PC followed by B1P1 against the control.

#### Quantitative Estimation of H_2_O_2_

**Figure 7:**
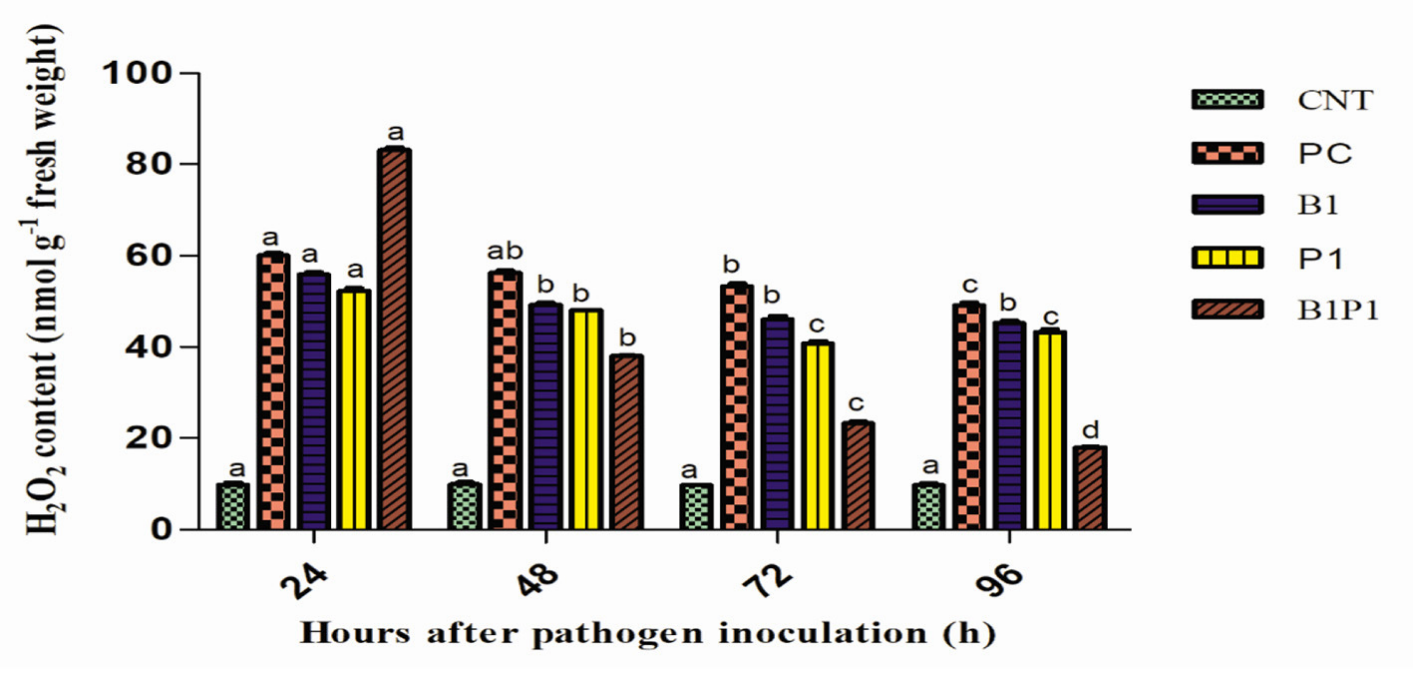
The H_2_O_2_ content in tomato leaves treated with different treatments from 24 to 96 h after pathogen inoculation. Data are shown as the mean ± SE (*n* = 3). Tukey's B *post-hoc* test at 5% confidence level (*p* < 0.05) was used to test the significance of the data. These alphabets denoted the different significant level.

Hydrogen peroxide content was quantified at different hours of pathogen infection. Initially, at 24 h, the H_2_O_2_ content increased sharply in all pathogen-treated plants. However, after 48 h, there was a steady decline in the H_2_O_2_ production (Figure 7) in all the treatments. At 48 h, the maximum H_2_O_2_ production was reported in PC (5.7-fold) increase and least in B1P1 (3.85-fold). At 72 h, the trend was the same but the fold increase was lowered in B1P1 (2.39-fold) and PC (5.49-fold) compared to the control.

#### ROS Determination Through ${\text{O}}_{2}^{-}$

**Figure 8:**
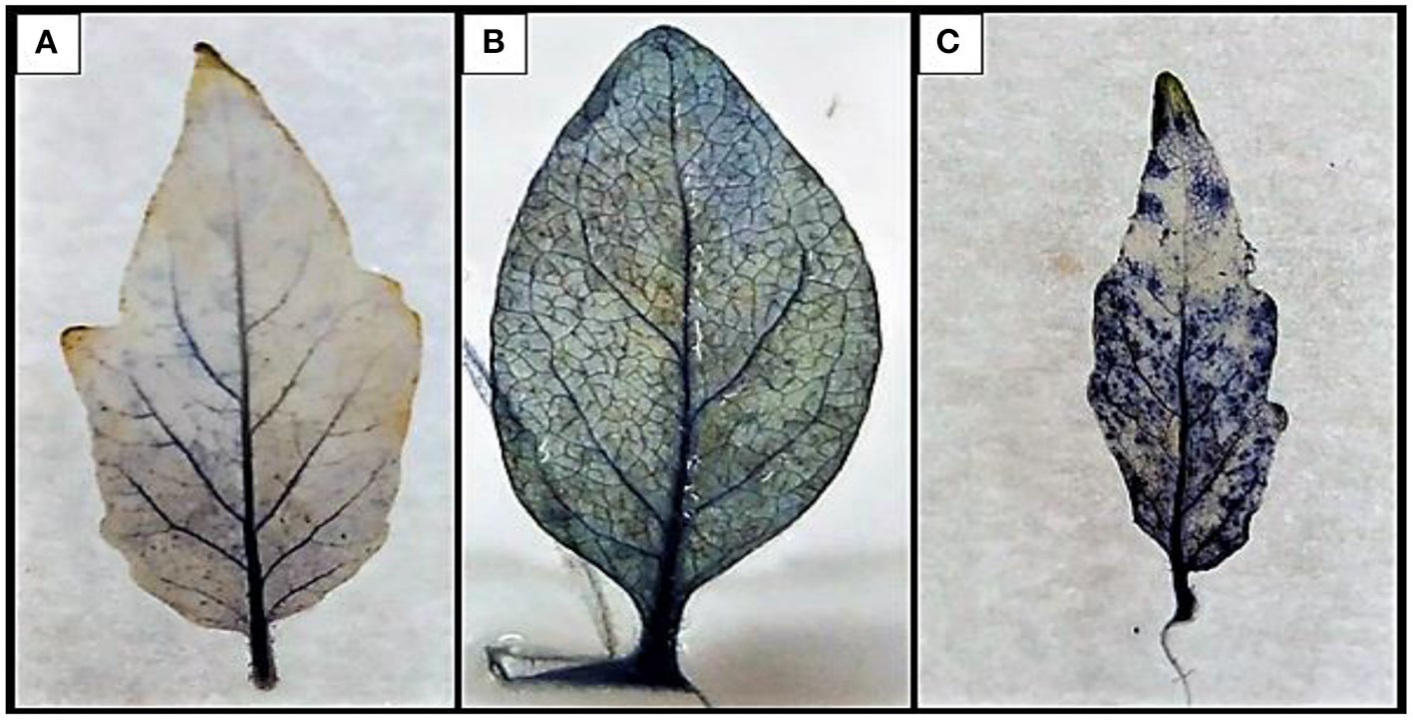
The development of blue-to-violet-colored formazan on the petiolar and in the interveinal part of leaf by the reduction of tetrazolium dye. **(A)** Control, **(B)** B1P1, and **(C)** PC.

Qualitative estimation of ROS production was evaluated through the generation of ${\text{O}}_{2}^{.-}$radical in the treated plant. The NBT reduction method was employed for the study upon the reduction of NBT, purple formazan was formed in the laminar and petiolar regions. The highest deposition was observed in the PC and the lowest in the B1P1 and the control (Figure 8).

#### Ascorbic Acid Assay

**Figure 9:**
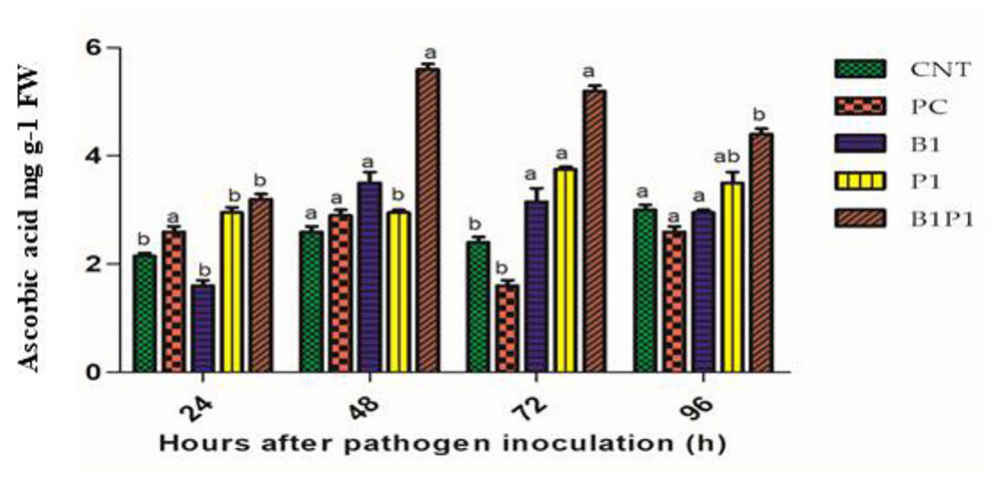
The AA content of tomato leaves treated with different treatments of 24–96 h of pathogen inoculation. Data are shown as a mean ±SE (*n* = 3). Tukey's B *post-hoc* test at 5% confidence level (*p* < 0.05) was used to test the significance of the data. These alphabets denoted the different significant level.

Ascorbic acid content was assayed for different treatments at different time intervals. At 48 h, the highest activity was recorded for B1P1 (2.8-fold increase) and the lowest for PC (1.11-fold increase) compared to the control. At 72 h, the AA accumulation showed the highest increase for B1P1 (3.25-fold) and the lowest in the PC compared to the control (Figure 9).

### Superoxide Dismutase Activity and GR Activity

**Figure 10:**
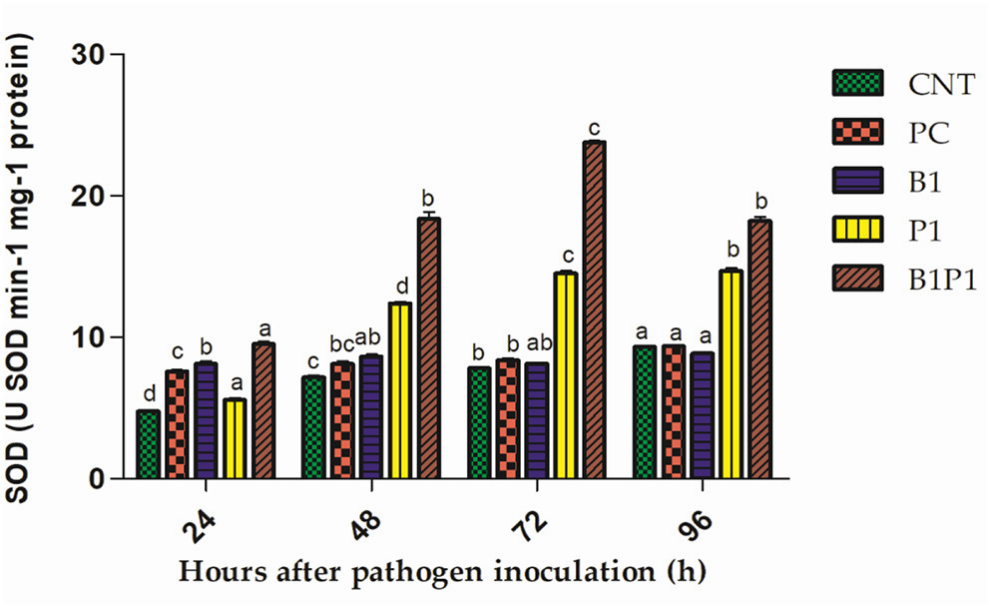
The SOD activity of tomato leaves treated with different treatments of 24–96 h of pathogen inoculation. Data are shown as mean ± SE (*n* = 3). Tukey's B *post-hoc* test at 5% confidence level (*p* < 0.05) was used to test the significance of the data. These alphabets denoted the different significant level.

**Figure 11:**
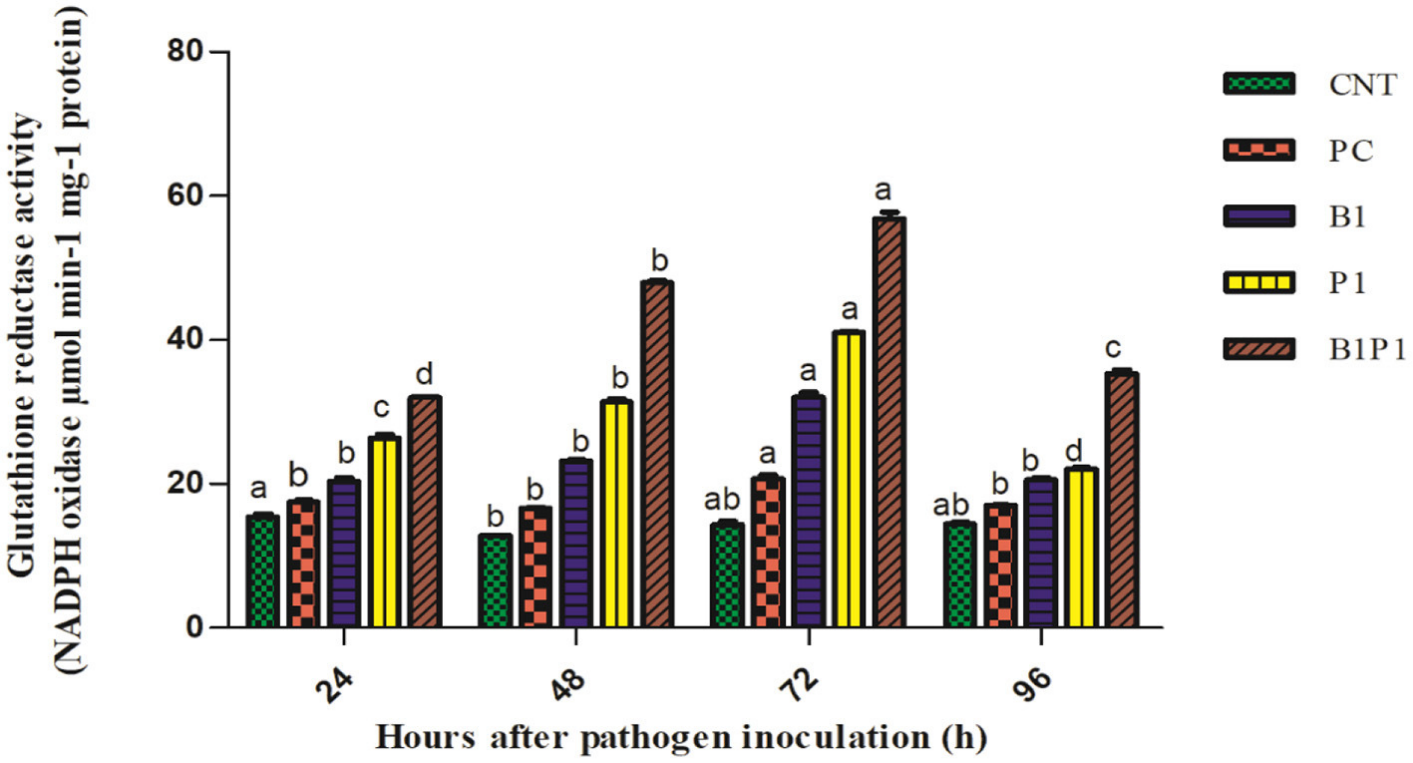
The GR activity of the tomato leaves treated with different treatments of 24–96 h of pathogen inoculation. Data are shown as a mean ± SE (*n* = 3). Tukey's B *post-hoc* test at 5% confidence level *(*p < 0.05) was used to test the significance of the data. These alphabets denoted the different significant level.

Superoxide dismutase and GR activity in the tomato plants increased from 24 to 72 h, and then decreased up to 96 h in pathogen-treated plants. The highest activity was observed at 72 h in B1P1 (3.05-fold increase) and the lowest in PC (1.07-fold increase) compared to the control plants in SOD (Figure 10). In GR, the highest activity was recorded at 72 h in B1P1 (3.96-fold increase) and the lowest in PC (1.44-fold increase) compared to the control (Figure 11).

### Phenylalanine-Ammonia Lyase Activity and Ascorbate Peroxidase Activity

**Figure 12:**
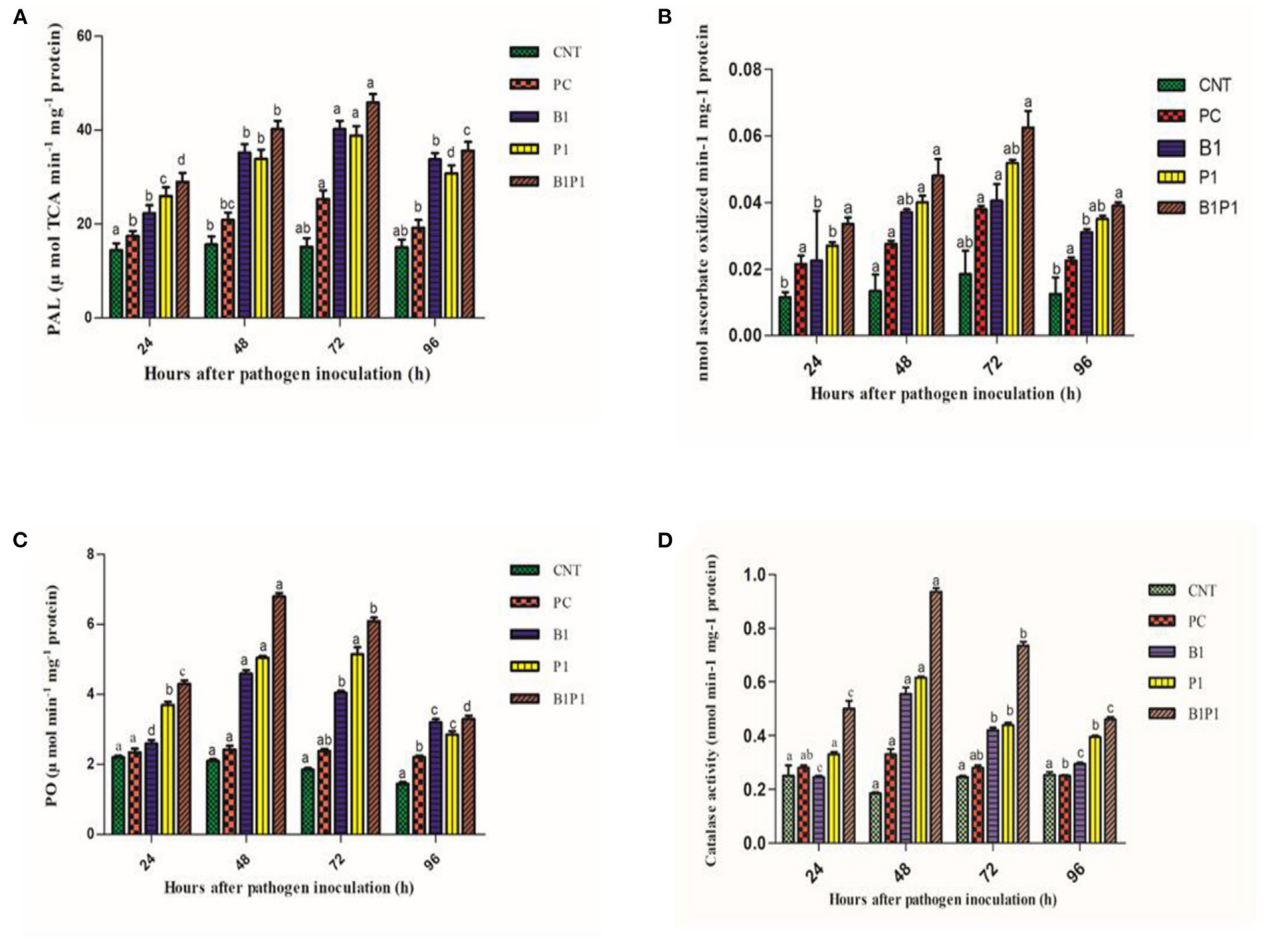
Enzymatic activity [PAL, **(A)**; APX, **(B)**; PO, **(C)**; CAT, **(D)**] of tomato leaves treated with different treatments of 24 h−96 h of pathogen inoculation. Data are shown as a mean ± SE (*n* = 3). Tukey's B *post-hoc* test at 5% confidence level (*p* < 0.05) was used to test the significance of the data. These alphabets denoted the different significant level.

Phenylalanine-ammonia lyase and APx activity increased from 24 to 72 h, and then decreased. All pathogen-treated plants showed alleviated values but the maximum was recorded at 72 h for B1P1 (3.23-fold increase) and the minimum for PC (1.89-fold increase) in the PAL (Figure 12). The APx showed a 1.9-fold increase in PC and a 4.4-fold increase in B1P1 (Figures 12A,B).

### Peroxidase

Peroxidase activity was found to increase from 24 to 48 h, but after 48 h, a decline was noted that lasted until 96 h. The highest POX activity was observed at 48 h for B1P1 (3.23-fold increase) and the lowest for PC (1.15-fold increase) compared to the control plant (Figure 12C).

### Catalase Activity

The catalase activity was assessed for various treatments. The activity increased up to 48 h, but thereafter decreased until 96 h. The highest activity was recorded at 48 h for B1P1 (5.05-fold increase) and the lowest for PC (1.11-fold increase) compared to the control (Figure 12D).

### Analysis of Variance

An analysis of variance was performed using two factors, i.e., ICs and ET, as well as their cumulative interactions, which were significant for all. Almost all factors and their interactions were found to be significant, and the double interaction (IC × ET) showed significance, from the highest to the lowest, for chlorophyll a, chlorophyll b, total chlorophyll, Fv/Fm, AA, H_2_O_2_, PAL, SOD, GR, CAT, POX, and APX, it showed comparatively less significance (Table 1).

**Table 1:** Variance analysis was done to examine the effect of ICs, ET, and their cumulative effect on chlorophyll a, chlorophyll b, total chlorophyll, Fv/Fm, AA, H_2_O_2_, SOD, PAL, CAT, POX, and APX (***p < 0.001; **p < 0.01).

**Parameters/variables**	**Inoculation**	**Evaluation**	**IC × ET**
	**conditions (IC)**	**time (ET)**	
Chlorophyll a	***	***	***
Chlorophyll b	***	***	***
Total chlorophyll	**	**	**
Fv/Fm	***	***	***
Ascorbic acid	***	***	***
H_2_O_2_	***	***	***
PAL	***	***	***
SOD	***	***	***
CAT	***	***	***
POx	***	***	***
APx	**	**	**
GR	***	***	***

### Principal Component Analysis

**Figure 13:**
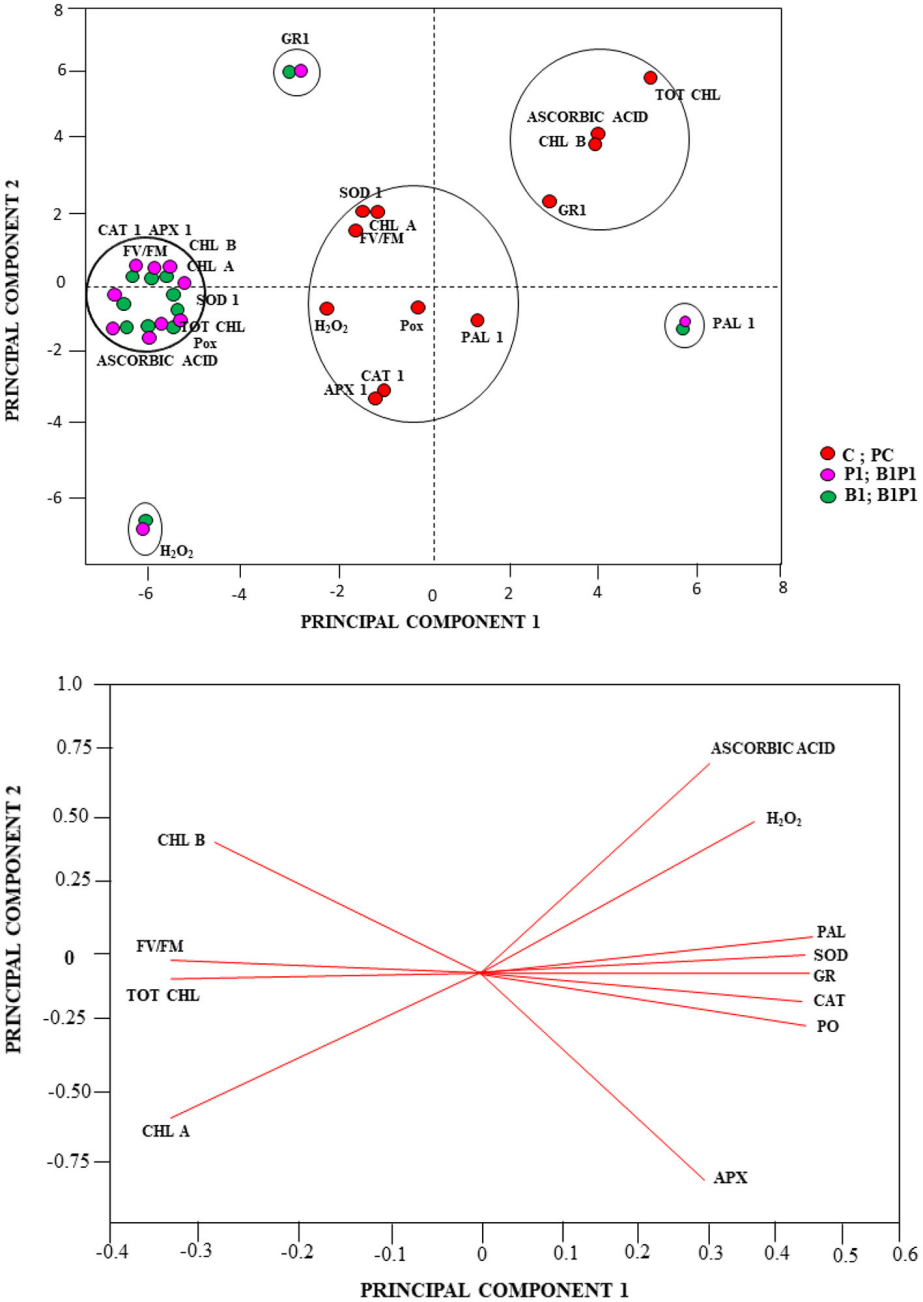
PCA analysis **(A)**, Score plot and **(B)**, loading plot of the PCA linking the total chlorophyll (tot chl), chlorophyll b (chl b), chlorophyll a (chl a), chlorophyll a fluorescence parameter (maximum PSII quantum efficiency (Fv/Fm), H_2_O_2_, AA, peroxidase (POX1), ascorbate peroxidase (APX1), catalase (CAT1), superoxide dismutase (SOD1), glutathione reductase (GR1), and phenylalanine-ammonia lyase (PAL1) of different treatments.

Besides the univariate and multivariate analysis, data were further subjected to PCA to gain more insights on the role played by the various treatments under different time intervals (24, 48, 72, and 96 h). The effects were shown with regard to biochemical and physiological changes using the first two PCs, which covered most of the variation of the dataset (PC1 and PC2 explained 81 and 7% of the total variance, respectively). The PCA revealed the effect of the various biochemical and physiological conditions of the treatments. Based on the Eigen analysis of correlation, the treatments clustered themselves in different quadrants of PCA depending on their values of similarities and dissimilarities. Therefore, to better understand the relationships, a multivariate PCA was carried out on the similarities and dissimilarities of the results of the biochemical and physiological traits. The multivariate PCA reduces a large number of variables to only a few, which accounts for the majority of the variance in the observed experimental results. Other treatments showed different variations with respect to various parameters, such as CAT, APX, chlorophyll a, chlorophyll b, total chlorophyll, Fv/Fm, POX, SOD, and AA, which were clustered together for different treatments; GR1, H_2_O_2_, and PAL parameters were clustered together at different loci for different treatments in the PCA axis (Figure 13A). PC1 showed positive scores for AA, H_2_O_2_, PAL, SOD, GR, CAT, POX, and APX (which were clustered together) and a negative score for Chl a, Chl b, total Chl, and Fv/Fm (Figure 13B).

### Colonization of Tomato by Plant Growth-Promoting Bacteria Against Pathogen

**Figure 14:**
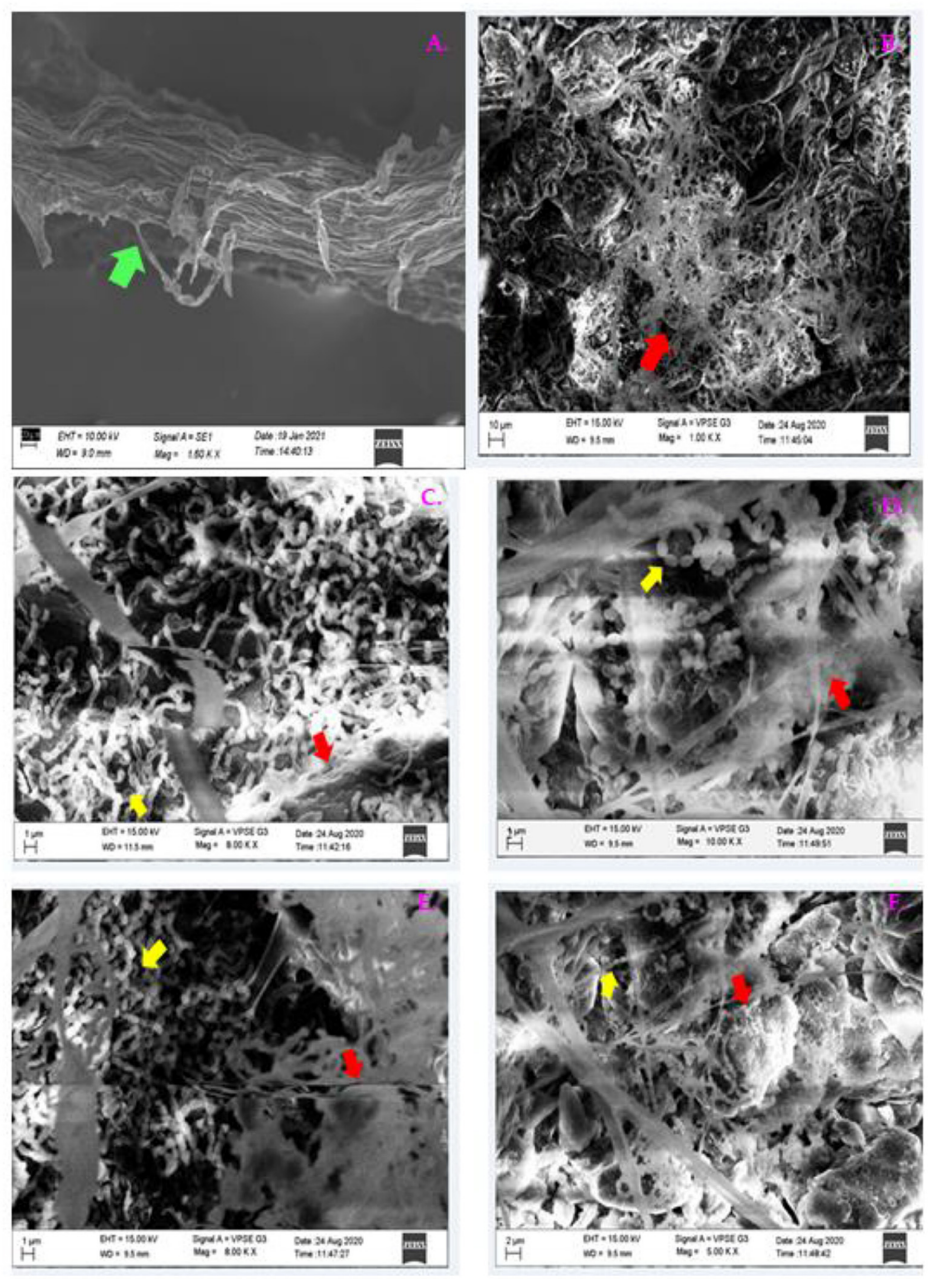
The SEM micrographs of plant roots colonized by B1 and P1 on the tomato roots after 48 h of pathogen invasion. Images were taken at the root tips; **(A)** CNT, **(B)** PC, **(C)** B1 at 1 μm; **(D)** B1 at 2 μm, **(E)** P1 at 1 μm; **(F)** P1 at 2 μm (Green arrow indicates root; red arrow shows pathogen invasion; and the yellow arrow indicates bacterial colonization).

Two days after pathogen invasion, the root colonization of PGPB around the tomato roots was studied with SEM. Root surface inoculated with B1 (Figures 14C,D) and P1 (Figures 14E,F) was examined; the images showed that the degree of colonization by a bacterial cell of isolates was consistent by forming dense microcolonies on the root surface around the pathogen showing good colonizing abilities. The bacterial cells were found closely attached to the root surface around the pathogen in bacterized seedlings in both the cases. Epidermal root surface in case of treated seedlings without bacterial inoculants (control) (Figure 14A). A dense floccose mycelium around the root surface was observed (Figure 14) in all the treatments except CNT. Figure 14B shows treatment PC.

## Discussion

*Sclerotium rolfsii* is a group of saprophytic and ubiquitous fungus that affects nearly 500 plant species and is considered to be one of the most devastating among fungal groups (Aycock, 1966; Motlagh and Kaviani, 2008). Plants respond to various biotic stresses by activating their antioxidative mechanisms, which is short-lived, and thus, provide an opportunity to pathogens to invade into the plant system; however, various rhizosphere colonists could prolong this effect (Lichtenthaler, 1987). In the present study, we have emphasized the effectiveness of using the microbial consortium-treated plants (*Bacillus subtillis, P. fluorescens*) to control the spread of pathogens in the tomato plant to evaluate the metabolic changes in tomato on *S. rolfsii* invasion. The BCAs are known to produce secondary metabolites and enzymes that help in obstructing the pathogen penetration, proliferation, and establishment into the hosts (Yadav et al., 2021). Plants when exposed to pathogen, physiological and biochemical alterations, were noticed in host systems, such as photosynthesis, translocation, growth, development, transpiration, and respiration, and a plant's antioxidant mechanism gets activated; and thus, the plant releases enzymes as a countermeasure. Hypersensitive cell death (HR) is triggered by ROS generation and stops the attack of obligate parasites (Apel and Hirt, 2004); and therefore, this could ease *S. rolfsii* throughout pathogenesis. To counteract the harmful effects of ROS, the plants are blessed with a diverse row of antioxidant enzymes (Kumudini et al., 2017).

The photosynthetic mechanism was evaluated for different treatments at different time intervals. Chlorophyll a, chlorophyll b, and total chlorophyll content was the highest in untreated plants and the microbial consortium-treated plants (B1P1), and the content was the lowest in PC plants, which reveals lesser channeling of light energy to the reaction center, and therefore, the highest deterioration of photosynthetic pigments in the PC plants. A reduction in the photochemical quenching done by chlorophyll fluorescence measurements (Fv/Fm) were due to PSII injury. The Fv/Fm was measured for different treatments at different time intervals, with the lowest value in PC plants followed by the microbial consortium-treated plants compared to the control. After successful recognition of the pathogen into the host system, a high accumulation of H_2_O_2_ and superoxide anion was recorded, which was further confirmed *via* histochemical analysis through the DAB staining method and NBT reduction method, respectively. Herein, the presence of dark brown tinct and purple formazan over the leaves confirmed the possible area of H_2_O_2_ and superoxide radical accumulation which was the highest in PC and the lowest in B1P1 compared to the untreated control plants. High accumulation of H_2_O_2_ leads to a reduction in the photosynthetic mechanism, and therefore, accelerates senescence in the host. The H_2_O_2_ is self-reliant in causing changes in the host cell wall *via* peroxidase-catalyzed cross-linking of polymers, such as proteins (Brisson et al., 1994). The oxidative burst and the production of H_2_O_2_ and O^2−^ showed an early defense response in the plants against several phytopathogens (Lubaina and Murugan, 2013).

Plants generally safeguard themselves from biotic and abiotic stresses through multiple chain antioxidative defense enzymes (Kapoor et al., 2015). The SOD dispenses the first line of defense against the oxidative stress, which causes dismutation of ${\text{O}}_{2}^{-}$ into H_2_O_2_ and O_2_. These harmful by-products are converted into H_2_O and ${\text{O}}_{2}^{-}$ by antioxidative enzymes, such as CAT and APX (Radwan, 2012). The SOD was reported to be the highest in *Mycosphaerella fragariae*-infected leaves as well as in resistant strawberry varieties (Ehsani-Moghaddam et al., 2006). In the present study, the SOD was found to be the highest in B1P1 and the lowest in PC compared to the control plants. The APX and CAT are two key enzymes that cause scavenging of produced H_2_O_2_. The CAT activity was recorded from 24 h to 96 h, but the highest activity was recorded at 48 h, with B1P1 showing the highest activity and PC showing the lowest activity compared to the control. The CAT activity increased up to 48 h, which indicates an active cleaning of H_2_O_2_ from the system, but after that, a reduction was recorded due to excessive production. Accretion of H_2_O_2_ into the cell and the abatement in CAT activity were supposed to increase because of proteolysis by peroxisomal endopeptidases, caused by oxidative stress, which leads to cellular degeneration (Mittler et al., 2004). It also protects the cells against lipid peroxidation (Karanastasi et al., 2018). The APx is considered an essential component of the ascorbate-glutathione cycle, which reduces H_2_O_2_ into H_2_O during oxidative stress and is responsible for confiscating undue H_2_O_2_ from mitochondria, chloroplast, and peroxisomes (Foyer et al., 1997). In our experiment, the APX increases in the microbial consortium-treated plants, followed by singly inoculated and PC compared to the control. Comparatively higher CAT activity in our experiments could be correlated with the fact that CAT does not need any reducing equivalents for its utility, such as APx, which marks it unresponsive to the redox status of cells; thus, it stays unaffected during unfavorable conditions. It is generally known that for correct scavenging of ROIs, high reduced peroxidized ratio of GSH and AA is imperative; thus, for excessive removal of H_2_O_2_, another antioxidative pathway, i.e., the ascorbate-glutathione cycle, comes into play (Asada, 2019). This involves a series of oxidation-reduction reactions comprising APX, GR, dehydroascorbate reductase (DHAR) and monodehydroascorbate reductase (MDHAR) (Brueske, 1980). In our study, higher GR and AA were the highest for B1P1 and the lowest for PC compared to uninoculated control plants. The POX is a heme-containing antioxidative enzyme that plays a major role in the polymerization of lignin through the phenyl-propanoid pathway (Nakano and Asada, 1981). In our study, POx was the highest in B1P1 and the lowest in PC at 48 h of pathogen outbreak, demonstrating the defense response provided by the microbial consortium-treated plants under stress. Conversion of L-phenylalanine into ammonia and trans-cinnamic acid is mediated by the PAL enzyme and it is the first and steadfast step in the phenylpropanoid pathway involved in the biosynthesis of polyphenols in plants as a defense barrier, thus shielding plants from stress. Here, we found the highest PAL activity in B1P1 and the lowest in PC at 72 h compared to the control plants. The imbalance between H_2_O_2_ production and scavenging enzyme pool is a strategic factor to resolve pathogen success and host captivating strategy, which reflects the host defense mechanism in tomato or the pathogenic approach of the fungus (Li et al., 2015; Kumar et al., 2021). Furthermore, the results obtained were subjected to PCA analysis to see the synergistic effect of time and treatments on the biochemical and physiological parameters, as they clustered themselves in various quadrantes depending on the results acquired through variance (Fagundes-Nacarath et al., 2018). The SEM analysis showed the strong colonization of B1 and P1 around the root epidermal indicating the potentiality of BCAs in controlling the pathogen invasion into the host.

Based on the above results, we can conclude that rhizosphere microbes develop strong stimuli in plants under stress; however, they also control ROS generation, and thus, have phytotoxic consequences. The enhanced response of all of these activities in the (*P. fluorescences* and *B. subtilis*) microbe-consortium treatment compared to their single application demonstrated synergistic behavior of the dual microbial consortium at reduced individual microbial load, which is positively correlated with reduced plant mortality. Thus, the use of microbial consortium in agricultural crops may be used to boost host defense responses against an invading pathogen.

## Conclusion

This study confirms the beneficial role of microbial consortium in alleviating the biotic stress in the tomato plants against *S. rolfsii*, one of the most damaging soil-borne phytopathogens. Priming with rhizosphere microbes induced the oxidative stress significantly and improved the activities of the defense enzymes against the attacking pathogen. The ROS generation is, however, considered a key indicator of molecules when establishing plant-pathogen interactions. Using rhizosphere microbes is an economically and eco-friendly alternate practice in managing plant diseases in an integrated manner, and this elicits an early release of antioxidative enzymes, as the first-line defense in plantpathogen interactions. The studied inoculum constitutes a promising method to reduce biotic stress. Basically, our findings confirm the beneficial role of the microbial consortium in alleviating the biotic stress in tomato plants against *S. rolfsii*, one of the most damaging soil-borne phytopathogens. The present results confirm the amelioration of biotic stress on physiochemical parameters under different treatments at different time intervals. Hence, the use of multifarious plant growth-promoting bacteria with feather-degraded biotic tolerant properties holds a great potential to be used as biocontrol agents for soil-borne phytopathogens. This study highlights a beneficial bipartite plant–microbe interaction between the tomato plant and biocontrol agents under short-term biotic stresses. Taken together, our results indicate that the biotic stress and the amelioration capacity of the tomato plant have been significantly improved with BCAs under all the treatments. Stress-induced symptoms in the host were significantly improved in the presence of BCAs. However, the role of BCAs used singly or with the microbial consortium in improving the stress tolerance/defense genes of the tomato plants at subsequent developmental stages can be an interesting topic for further investigation. From the result of the present study, it can be concluded that the addition of a rhizobacterium with multifarious plant growth-promoting properties act synergistically to mitigate the pathogen-induced damage and enhance the biotic tolerance in the tomato plants. Application of *B. subtilis* and *P. fluorescences* also restores membrane integrity by minimizing ROS-generated oxidative damage and promotes plant growth by augmenting the induced systemic resistance to cope with biotic stress responses. The test isolate increases the anti-oxidative defense mechanism under biotic stress, which suggests the induction of a systemic response and provides a valuable insight of microbial-mediated enhanced tolerance against the pathogen.

## Data Availability Statement

The original contributions presented in the study are included in the article/supplementary material, further inquiries can be directed to the corresponding author/s.

## Ethics Statement

VS, the undersigned, give her consent for the publication of identifiable details, which can include photograph(s) and/or videos and/or case history and/or details within the manuscript to be published in the above Journal and Article.

## Author Contributions

The experiments were planned and directed by RU and most of the experiments were executed by VS. Manuscript preparation was done by RU and VS. Calculations were performed by SK and YT. All authors read and helped in finalizing the final manuscript and have read and agreed to the published version of the manuscript.

## Conflict of Interest

The authors declare that the research was conducted in the absence of any commercial or financial relationships that could be construed as a potential conflict of interest.

## Publisher's Note

All claims expressed in this article are solely those of the authors and do not necessarily represent those of their affiliated organizations, or those of the publisher, the editors and the reviewers. Any product that may be evaluated in this article, or claim that may be made by its manufacturer, is not guaranteed or endorsed by the publisher.

## Abbreviations

ROS: reactive oxygen species
SOD: superoxide dismutase
POx: peroxidase
PAL: phenylalanine-ammonia lyase
CAT: catalase
GR: glutathione reductase
APx: ascorbate peroxidase
CTAB: Cetyl trimethyl ammonium bromide
PDA: potato dextrose agar
CMC: Carboxy-methyl cellulose
NBT: nitroblue tetrazolium
SDW: sterilized distilled water
DAB, 3: 3′-Diaminobenzidine
PCA: principal component analysis.
